# Antifungal Therapy for Systemic Mycosis and the Nanobiotechnology Era: Improving Efficacy, Biodistribution and Toxicity

**DOI:** 10.3389/fmicb.2017.00336

**Published:** 2017-03-07

**Authors:** Ana C. O. Souza, Andre C. Amaral

**Affiliations:** ^1^Laboratory of Pathogenic Dimorphic Fungi, Institute of Biomedical Sciences, University of São PauloSão Paulo, Brazil; ^2^Laboratory of Nano and Biotechnology, Institute of Tropical Pathology and Public Health, Federal University of GoiásGoiânia, Brazil

**Keywords:** antifungal therapy, fungal infection, mycosis, nanobiotechnology, drug delivery systems

## Abstract

Fungal diseases have been emerging as an important public health problem worldwide with the increase in host predisposition factors due to immunological dysregulations, immunosuppressive and/or anticancer therapy. Antifungal therapy for systemic mycosis is limited, most of times expensive and causes important toxic effects. Nanotechnology has become an interesting strategy to improve efficacy of traditional antifungal drugs, which allows lower toxicity, better biodistribution, and drug targeting, with promising results *in vitro* and *in vivo*. In this review, we provide a discussion about conventional antifungal and nanoantifungal therapies for systemic mycosis.

## Introduction

Fungal diseases have arisen as an important public health problem worldwide, having a great impact in human morbidity and mortality, specially among immunocompromised individuals (Denning and Hope, 2010; Shapiro et al., 2011; Caffrey and Obar, 2016; Prasad et al., 2016). Although superficial mycosis are the most common among fungal infections, affecting nearly 25% of the human population worldwide (Havlickova et al., 2008), invasive fungal infections are of greater concern, since they are life-threatening, difficult to diagnose and account with a limited number of therapeutic options (Brown et al., 2012a,b). It is estimated that systemic mycosis cause about 1.5 million deaths annually (Brown et al., 2012a; Caffrey and Obar, 2016).

Systemic fungal infections include both opportunistic and endemic mycosis, and are associated with high rates of mortality if not readily diagnosed and treated (Brown et al., 2012a; Caffrey and Obar, 2016). Opportunistic infections are caused by environmental or commensal fungi and affect immunocompromised or genetically predisposed hosts, accounting for about 2 million life-threatening reported cases each year worldwide (Brown et al., 2012a; Polvi et al., 2015; Caffrey and Obar, 2016). Species from genera *Aspergillus, Candida, Cryptococcus*, and *Pneumocystis* are responsible for more than 90% of all reported fungal-related deaths, although accurate incidence data is not officially available and may be underestimated (Brown et al., 2012a,b).

On the other hand, endemic mycoses are caused by thermal-dimorphic fungi and can affect especially immunocompetent hosts that live in particular geographic areas, although immunosuppression is a risk factor and contributes to the outcome of infection (Goughenour and Rappleye, 2017). Dimorphic fungi occur as saprophytic molds in the environment. After inhalation or trauma inoculation by the mammalian host, they transform into pathogenic yeasts or spherules (Bonifaz et al., 2011; Sil and Andrianopoulos, 2015). Among the causative agents of endemic mycosis are *Histoplasma capsulatum, Coccidioides immitis* and *C. posadasii, Paracoccidioides brasiliensis*, and *P. lutzii, Blastomyces dermatitidis, Talaromyces marneffei*, and *Sporothrix schenckii*. Despite incidence data of infections by dimorphic fungi are usually inaccurate due to under-diagnosis and under-reporting, it is believed that endemic mycosis are responsible for approximately 65,000 life-threatening cases each year worldwide (Brown et al., 2012a; Goughenour and Rappleye, 2017).

The fact that fungi and animals are evolutionarily close makes the search for therapeutic targets a big challenge, since targets such as biomolecules synthesis have great potential for toxicity (Groll et al., 1998; Denning and Hope, 2010; Heitman, 2011; Shapiro et al., 2011; Polvi et al., 2015). Today, only a dozen of antifungal agents (Table 1) are approved for the treatment of invasive fungal infections (Seyedmousavi et al., 2017). Antifungal therapy for systemic mycosis is basically focused in three classes: polyenes, azoles, and echinocandins (Polvi et al., 2015). Therapies for invasive infection presents restrictions such as route of administration, toxicity, drug interactions and sometimes high costs, considering patients hospitalization (Denning and Hope, 2010; Brown et al., 2012b; Polvi et al., 2015). In some cases, prolonged treatment times are needed, together with clinical interventions due to side effects (Goughenour and Rappleye, 2017). Recently, drug resistance has also become a worrisome issue (Xie et al., 2014). Unfortunately, antifungal drug development does not follow the progressive increase of invasive infections resulted from modern medical interventions, primary, and acquired immunodeficiencies and immunosuppressive therapies. Besides, there are no approved human vaccines for fungal diseases at this moment (Brown et al., 2012b). To overcome these problems, it is interesting to develop cheaper and novel therapeutic strategies in the battle against fungal diseases.

**Table 1:** **Current antifungal agents available for the therapy of systemic mycosis**.

**Antifungal spectrum**	**AMB**	**5FC**	**FLU**	**ITR**	**VOR**	**POS**	**ISA**	**CAS**	**MIC**	**ANI**
*Candida albicans*	++	++	++	++	++	++	++	++	++	++
*Candida glabrata*	++	++	+	+	++	++	++	+	+	+
*Candida parapsilosis*	++	++	++	++	++	++	++	++	++	++
*Candida tropicalis*	++	++	++	++	++	++	++	++	++	++
*Candida krusei*	++	+	–	+	++	++	++	++	++	++
*Candida lusitaniae*	–	++	++	++	++	++	++	++	++	++
*Aspergillus fumigatus*	++	–	–	+	++	++	++	+	+	+
*Cryptococcus neoformans*	++	++	++	++	++	++	++	–	–	–
Mucorales	++	–	–	–	–	++	++	–	–	–
*Fusarium* spp.	+	–	–	+	++	++	++	–	–	–
*Scedosporium* spp.	+	–	–	+	+	+	+	–	–	–
*Blastomyces dermatitidis*	++	–	+	++	++	++	++	–	–	–
*Coccidioides immitis*	++	–	++	++	++	++	++	–	–	–
*Histoplasma capsulatum*	++	–	+	++	++	++	++	–	–	–
**Class**	Polyene	Pyrimidine	Azole					Echinocandins		
**Target**	Ergosterol	Nucleic acid	Ergosterol					Cell wall		
**Administration**	Intravenous	Oral	Oral/Intravenous					Intravenous		
**Side Effects**	Infusion reactions, hepatotoxicity, nephrotoxicity	Bone marrow suppression, liver toxicity	Gastrointestinal upset, hepatotoxicity, liver failure					Infusion reactions, gastrointestinal upset, headache, liver toxicity		

*5FC, flucytosine; AMB, amphotericin B; ANI, anidulafungin; CAS, caspofungin; FLU, fluconazole; ISA, isavuconazole; ITR, itraconazole; MIC, micafungin; POS, posaconazole; VOR, voriconazole*.

*Adapted from Nett and Andes (2016)*.

In this review, we will discuss current antifungal therapies available for systemic infections and point out some of the strategies using nanobiotechnology to improve conventional therapy. We will give a brief introduction about nanoformulations, and provide an overview of current studies proposing nanostructuration as an approach to improve efficacy and bioavailability of conventional antifungal drugs.

## Conventional therapy for invasive fungal diseases

In the late 1950s, polyenes emerged as the first class of antifungal agents. These molecules are produced by *Streptomyces nodosus* presenting high affinity for ergosterol, the major sterol in fungal cell membrane, which is responsible for membrane fluidity, asymmetry, and integrity (Odds et al., 2003; Carrillo-Muñoz et al., 2006; Mesa-Arango et al., 2012). By binding to ergosterol, polyene molecules complex forming pores that destabilize cell membrane, allowing leakage of cellular contents and resulting in fungal cell death (Finkelstein and Holz, 1973; Georgopapadakou, 1998; Mesa-Arango et al., 2012; Adler-Moore et al., 2016). Besides, induction of oxidative damage in the fungal cell also contributes to fungicidal activity (Georgopapadakou, 1998; Mesa-Arango et al., 2012). Unluckily, polyene agents can also interact with cholesterol, what confers potential toxicity for mammalian cells (Hsuchen and Feingold, 1973; Georgopapadakou, 1998; Mesa-Arango et al., 2012). Among Polyenes, Amphotericin B is the most used for the treatment of systemic fungal infections. Amphotericin B has fungicidal effects against a broad-spectrum of fungal pathogens and is approved for the treatment of numerous invasive mycosis, such as candidiasis, aspergillosis, cryptococcosis, blastomycosis, histoplasmosis, mucormycosis, and sporotrichosis (Georgopapadakou, 1998; Mesa-Arango et al., 2012; Adler-Moore et al., 2016; Nett and Andes, 2016).

Amphotericin B was first introduced in the market in 1958 as a sodium deoxycholate solution administrated by parenteral route (Bartner et al., 1958; Groll et al., 1998), and after almost 60 years, it is still considered the gold standard for the treatment of most life-threatening mycosis (Groll et al., 1998; Carrillo-Muñoz et al., 2006; Mesa-Arango et al., 2012). However, this formulation is associated with important acute and chronic side-effects, particularly nephrotoxicity (Carrillo-Muñoz et al., 2006; Laniado-Laborín and Cabrales-Vargas, 2009; Mesa-Arango et al., 2012; Nett and Andes, 2016). Amphotericin B has the ability to stimulate proinflammatory responses, enhancing antifungal activity, although this may also be associated with toxicity (Mesa-Arango et al., 2012). In order to circumvent toxicity problems, lipid formulations were developed, including liposomes (Ambisome®), lipid complexes (Abelcet®), and colloidal dispersions (Amphocil®/Amphotech®) (Georgopapadakou, 1998; Gulati et al., 1998; Dupont, 2002; Mesa-Arango et al., 2012; Nett and Andes, 2016). However, these alternatives can be up to 20-fold more expensive than sodium deoxycholate Amphotericin B (Georgopapadakou, 1998; Wong-Beringer et al., 1998; Dismukes, 2000; Falci et al., 2011), limiting its usage in public health systems with limited resources.

In the late 1960s, flucytosine (5-fluorocytosine), a synthetic pyrimidine analog originally designed for antitumor therapy, was first used in the treatment of invasive mycosis (Tassel and Madoff, 1968). After being imported to the fungal cell by cytosine permeases, 5-fluorocytosine is converted to fluorouracil, which gets incorporated into DNA and RNA molecules during their synthesis, inhibiting protein synthesis and DNA replication, thus impairing cell function (Groll et al., 1998; Nett and Andes, 2016; Prasad et al., 2016). These agents have activity against a limited spectrum of pathogenic yeasts, such as *C. albicans, C. glabrata, C. parapsilosis, C. tropicalis*, and *Cryptococcus* spp., and are poor effective against dimorphic or filamentous fungi (Nett and Andes, 2016; Prasad et al., 2016). Due to rapid occurrence of resistance during the therapy with flucytosine, specially among *Candida* species, its clinical use is preferable only in combination with other antifungal drugs, such as Amphotericin B in the treatment of cryptococcal meningitis and other life-threatening *Candida* infections (Tassel and Madoff, 1968; Francis and Walsh, 1992; Dismukes, 2000; Sanglard et al., 2009; Nett and Andes, 2016; Prasad et al., 2016). Besides, Flucytosine induces significant side-effects, like liver dysfunction and bone marrow suppression (Francis and Walsh, 1992; Groll et al., 1998; Dismukes, 2000; Nett and Andes, 2016; Prasad et al., 2016). In the United States, flucytosine is available in oral capsules (Groll et al., 1998; Nett and Andes, 2016).

Azoles are synthetic cyclic organic molecules introduced in the early 1970s in addition to antifungal arsenal. They are composed by a 5-member azole ring, which contains two (imidazoles) or three (triazoles) nitrogen atoms, attached to a complex side chain (Georgopapadakou, 1998; Groll et al., 2003). Azoles target is ergosterol biosynthesis, which is impaired due to inhibition of fungi cytochrome P-450 14-α sterol demethylase (Vanden Bossche et al., 1995; Georgopapadakou, 1998; Groll et al., 1998, 2003; Odds et al., 2003; Carrillo-Muñoz et al., 2006). As a consequence, cell membrane integrity is impaired, with sterol precursors accumulation inside fungal cell and depletion of ergosterol in cell membrane, altering normal permeability and fluidity (Georgopapadakou, 1998; Groll et al., 1998; Odds et al., 2003). In general, azoles have a fungistatic action, affecting cell growth and proliferation, and eventually, due to accumulation of toxic methylated sterols, fungal cell death may occur (Groll et al., 2003; Zonios and Bennett, 2008; Sanglard et al., 2009; Arnold et al., 2010; Shapiro et al., 2011; Prasad et al., 2016). Currently, azoles are the most diverse class of antifungal agents and they have been refined during the past 40 years (Odds et al., 2003). Imidazoles emerged first (Groll et al., 1998; Prasad et al., 2016), and among them, miconazole and ketoconazole were the only available for systemic use (Groll et al., 2003), with the last being the first orally absorbable antifungal and the first alternative to Amphotericin B (Groll et al., 1998; Seyedmousavi et al., 2017). Triazoles came next, Itraconazole in oral formulations, and Fluconazole, in both oral and i.v. formulations, both better tolerated and more effective than Ketoconazole (Dismukes, 2000), with increased potency, expanded antifungal spectrum and improved resistance to metabolic degradation (Como and Dismukes, 1994; Groll et al., 1998). Fluconazole has good activity against *Cryptococcus* spp., *Coccidioides* spp., and *Candida* spp. except for *C. krusei* and *C. glabrata* (Arnold et al., 2010; Denning and Hope, 2010; Nett and Andes, 2016; Seyedmousavi et al., 2017). Itraconazole has broader antifungal spectrum, being effective against *Candida* spp., *Cryptococcus neoformans, Aspergillus* spp., dimorphic fungi, and dermatophytes (Arnold et al., 2010; Denning and Hope, 2010; Nett and Andes, 2016).

Further modifications in the molecules gave rise to the second generation of triazoles, in which Voriconazole (structurally related to Fluconazole) and Posaconazole (related to Itraconazole) are available for systemic therapy with even better antifungal potency and specificity. Voriconazole spectrum of activity is improved in relation to first generation triazoles, also including *C. glabrata, Fusarium* spp., *Scedosporium* spp. (Arnold et al., 2010; Denning and Hope, 2010; Nett and Andes, 2016). Posaconazole exhibits the widest antifungal spectrum of the azoles, being active against both yeasts and molds, including several Mucorales species (Arnold et al., 2010; Denning and Hope, 2010; Nett and Andes, 2016). Isavuconazole is the newest triazole introduced in the market in 2015 and recently approved for the treatment of invasive aspergillosis and mucormycosis in the USA and Europe (McCormack, 2015).

Although azole antifungals are generally well-tolerated (Odds et al., 2003; Carrillo-Muñoz et al., 2006), they are substrates and inhibitors of several cytochrome P-450 enzymes, what is the mainly cause of their adverse effects, specially hepatotoxicity (Carrillo-Muñoz et al., 2006). For this reason, azoles can also impair metabolism of coadministered drugs, what leads to decreased plasma concentration and unexpected toxicity (Groll et al., 1998; Dismukes, 2000; Shapiro et al., 2011; Nett and Andes, 2016; Prasad et al., 2016). Besides, due to teratogenic effects, azoles are contraindicated during pregnancy (Arnold et al., 2010; Nett and Andes, 2016). Lastly, the emergence of resistance among fungal isolates is another limitation and one of the motivations for the improvement of this class of antifungals (Dismukes, 2000; Shapiro et al., 2011; Prasad et al., 2016).

Echinocandins are the newest category of antifungals agents, consisting in fungi derived semisynthetic lipopeptides composed of a cyclic hexapeptide core and a variable lipid side chain responsible for their antifungal activity (Groll et al., 2003; Odds et al., 2003). Although they were discovered in the 1970s, only 30 years later their use was approved by the US Food and Drug Administration. Echinocandins inhibits the synthesis of 1,3-β-glucan, a structural fungal cell wall polysaccharide that is responsible for cell wall's shape and rigidity, osmotic integrity and is important in cell division and cell growth (Georgopapadakou, 2001; Groll et al., 2003; Odds et al., 2003). Echinocandins includes caspofungin, anidulafungin and micafungin, all of them only available for i.v. administration (Groll et al., 2003; Odds et al., 2003). Their effect is species-dependent, acting as fungicidal against *Candida* spp. and fungistatic against *Aspergillus* spp. (Nett and Andes, 2016), with variable activity against dematiaceous and endemic mold (Seyedmousavi et al., 2017). However, echinocandins have no activity against *Scedosporium* spp., *Fusarium* spp., and *C. neoformans* (Groll et al., 2003; Odds et al., 2003; Arnold et al., 2010; Nett and Andes, 2016). Since echinocandins target β-glucan, which is not present in the mammalian cell, they present minimal side effects in humans (Groll et al., 2003; Sanglard et al., 2009; Arnold et al., 2010; Shapiro et al., 2011; Prasad et al., 2016), which may include gastrointestinal upsets headache and increased liver aminotransferases. Few drug-drug interactions were reported since echinocandins are not metabolized through cytochrome P-450 enzymes (Arnold et al., 2010; Nett and Andes, 2016).

**Figure 1:**
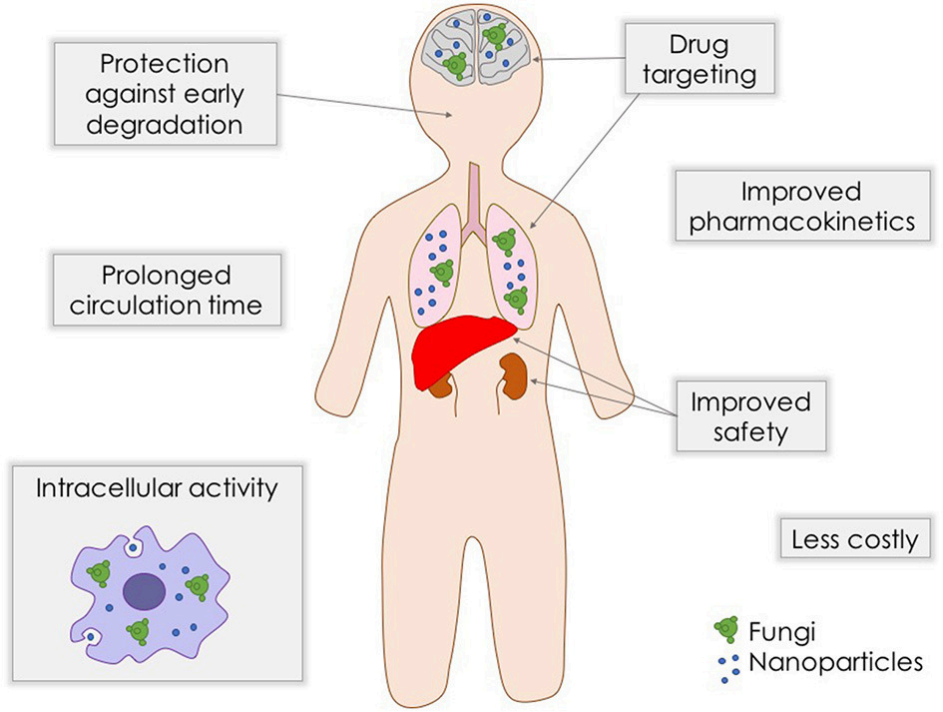
**Benefits of nanobiotechnology approaches for delivery of antifungal drugs**.

As stated above, although antifungal arsenal currently available for systemic mycoses is effective against the majority of fungal pathogens, they present limitations such as toxicity, drug-drug interactions, and emergence of clinical resistance. In order to overcome these problems, strategies like combination therapy (Mukherjee et al., 2005) and even repurposing of established medications (Butts and Krysan, 2012) are being exploited. The search for new compounds and new targets, together with the improvement of existing formulations are extremely needed. Nanostructuration of conventional antifungal agents may be an interesting alternative to achieve a better antifungal efficacy and safety (Figure 1).

## Nanobiotechnological approaches for antifungal delivery

**Figure 2:**
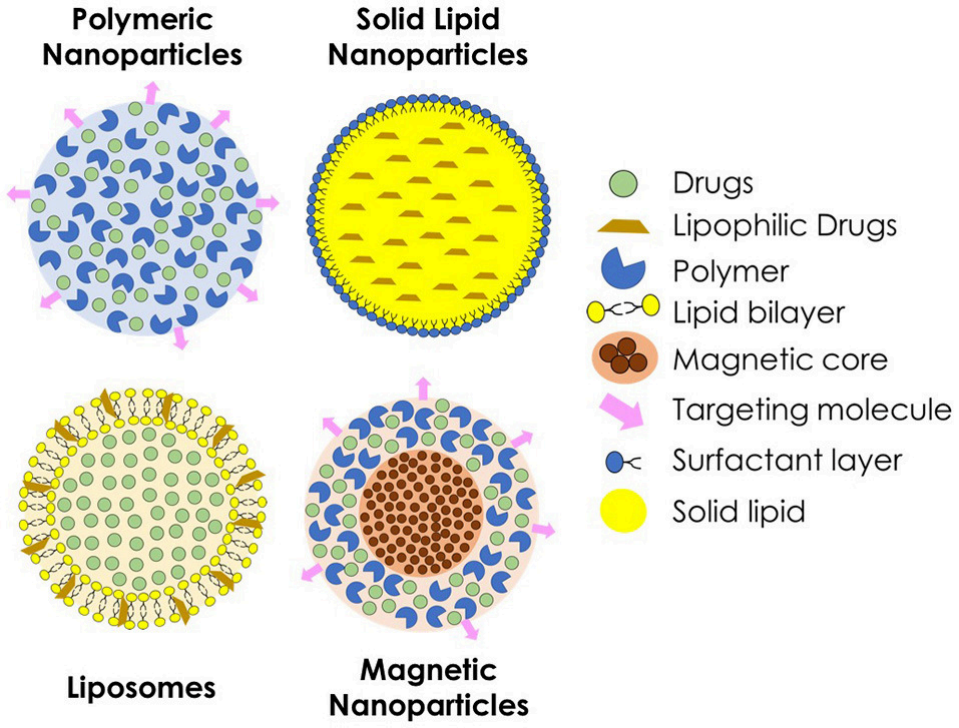
**Schematic representation of different types of nanoparticles used for the delivery of antifungal agents**.

Drug delivery systems containing nanoparticles have been object of intense investigation for the past decades, becoming an efficient strategy to increase drug bioavailability, reduce toxicity and enhance antifungal potency (Vyas and Gupta, 2006; Amaral and Felipe, 2013; de Sá et al., 2015; Stiufiuc et al., 2015). Among the advantages of this approach is the possibility to build formulations for a smart delivery, for example, targeting specific tissues and organs, such as the lungs, which are frequently the initial infection site during systemic fungal diseases (Amaral et al., 2009; Malathi and Balasubramanian, 2011; Moritz and Geszke-Moritz, 2015). In some cases, during intracellular infections, nanoparticles can penetrate the cells, leading the drug to act directly against the pathogen (Borborema et al., 2011; Dube et al., 2014). On the other hand, when coated with substances such as polyethylene glycol (PEG), they can evade recognition by the phagocytic system, preventing the attachment of opsonines, and promoting a longer circulation time (Stiufiuc et al., 2015). Studies have shown that nanoparticles also have desirable characteristics to be used as adjuvants in vaccines (Van Slooten et al., 2001; Agger et al., 2008; Bhowmick et al., 2008; Ribeiro et al., 2013). Currently, many types of nanostructures are under investigation for drug delivery of antifungal drugs, such as polymeric nanoparticles, solid lipid nanoparticles, liposomes, and magnetic nanoparticles (Figure 2).

Polymeric nanoparticles are prepared from various natural or synthetic polymers, both hydrophilic and hydrophobic (Pagels and Prud'homme, 2015). These polymers may be of natural origin, such as chitosan and alginate, or synthetics, such as poly (lactic acid, PLA) and poly (glycolic acid, PGA) or a combination of both forming the poly (lactide-*co*-glycolide acid, PLGA) (Bolhassani et al., 2014). When applied *in vivo*, polymeric nanoparticles suffer biodegradation to be metabolized and excreted by the organism, what confers better biocompatibility (Amaral and Felipe, 2013). As the polymer is degraded, the drug incorporated within is released to the medium and can efficiently reach the site of action.

Liposomes are particles made by natural or synthetic phospholipids. Because of the nature of these molecules, when in contact with an aqueous medium, they form spherical structures containing an aqueous nucleus surrounded by a lipid bilayer (Bozzuto and Molinari, 2015), which can be suitable for delivery of both hydrophilic and hydrophobic substances (Stiufiuc et al., 2015). Because of their sensitivity to pH variations, liposomes permeability can be adjusted, releasing the drug into specific sites, such as macrophage compartments with altered pH during an infection (Wang et al., 2014; Jiang et al., 2015). A successful example of this class of nanoparticles is the commercially available liposomal Amphotericin B, brand name Ambisome® (Reis, 2015). In addition, solid lipid nanoparticles have been intensively investigated, specially for topical drug delivery (Kumar and Sinha, 2016; Trombino et al., 2016). They are spherical nanoparticles constituted of physiological and biodegradable lipids, such as stearic acid. Due to chemical composition, solid lipid nanoparticles allow the nanostructuration of insoluble drugs, and have low toxicity.

Magnetic nanoparticles have received special attention for biological applications, mainly because of their ability to be manipulated by a magnetic field, so that it can be directed and delivered to, theoretically, a specific site of the organism (Hussein et al., 2014). This type of nanoparticles can be prepared by different chemical and physical methodologies using ferrous compounds such as cobalt, manganese and zinc ferrites, magnetite, and maghemite. In the medical field, these magnetic nanoparticles are mainly used for clinical diagnosis through magnetic resonance imaging (MRI). This type of nanoparticle can also be associated with other nanostructures, such as polymeric nanoparticles or liposomes, to combine characteristics for both MRI and drug delivery (Jain et al., 2008). The superparamagnetic iron oxide nanoparticles, a specific kind of magnetic nanoparticle, respond more efficiently to an external magnetic field (Kumar et al., 2010) presenting a great potential to be used as drug carriers.

There are over a 100 publications proposing new nanoformulations for antifungal drugs (Table 2). In this review, we will summarize the major findings for nanoparticles that had proven *in vivo* and/ or *in vitro* antifungal activity.

**Table 2:** **Examples of studies reporting antifungal nanoparticles with ***in vivo*** and/or ***in vitro*** activity**.

**Antifungal agent**	**Type Of nanoparticle**	**Administration**	***In vivo***	***In vitro***	**References**
Amphotericin B	Lipid nanoparticles	Inhalation	N/A	*Candida albicans, Cryptococcus neoformans*	Gangadhar et al., 2014
		Intravenous	*Aspergillus fumigatus*	*Candida albicans, Aspergillus fumigatus*	Jung et al., 2009
		Intravenous	*Aspergillus fumigatus*	N/A	Sheikh et al., 2010
		Intravenous	*Candida albicans*	N/A	Burgess et al., 2013
	Liposomes	N/A	N/A	*Candida albicans, Candida tropicalis*	Albasarah et al., 2010
	Magnetic nanoparticles	Nasal instilation	*Paracoccidioides brasiliensis*	N/A	Saldanha et al., 2016
	Nanoemulsion	Topical	N/A	*Aspergillus fumigatus, Aspergilus niger, Candida albicans*	Hussain et al., 2016
	Nanosuspension	Ocular	N/A	*Fusarium solani*	Das and Suresh, 2011
	Polymeric nanoparticles	Inhalation	*Aspergillus fumigatus*	N/A	Shirkhani et al., 2015
		Intraperitoneal	*Aspergillus fumigatus*	N/A	Van de Ven et al., 2012
		Intraperitoneal	*Paracoccidioides brasiliensis*	N/A	Amaral et al., 2009; Souza et al., 2015
		Intravenous	*Candida albicans*	N/A	Tang et al., 2014, 2015a
		Intravenous	*Candida glabrata*	N/A	Tang et al., 2015b
		Intravenous	*Cryptococcus neoformans*	N/A	Xu et al., 2011
		Intravenous	N/A	*Candida albicans*	Han et al., 2007
		Intravenous		*Candida albicans*	Zia et al., 2015
		N/A	*Candida glabrata*	N/A	Tang et al., 2015c
		N/A	N/A	*Candida albicans*	Tiyaboonchai et al., 2001; Tang et al., 2014
		N/A	N/A	*Candida albicans, Candida tropicalis*	Casa et al., 2015
		Ocular	N/A	*Candida albicans*	Zhou et al., 2013
		Ocular	N/A	*Candida albicans, Aspergillus fumigatus*	Zhou et al., 2013
		Oral	*Candida albicans, Aspergillus fumigatus*	N/A	Serrano et al., 2015
	Polymeric nanoparticles	Oral/intravenous	*Aspergillus fumigatus*	N/A	Italia et al., 2011
		Topical	*Candida albicans, Candida glabrata, Candida parapsilosis*	N/A	Sanchez et al., 2014
	Solid Lipid Nanoparticles	N/A	N/A	*Candida albicans*	Vieira and Carmona-Ribeiro, 2008
		Oral	N/A	*Candida albicans*	Chaudhari et al., 2016
Amphotericin B and Nystatin	Magnetic nanoparticles	N/A	N/A	*Candida albicans*	Niemirowicz et al., 2016
Fluconazole	Polymeric nanoparticles	Ocular	*Candida albicans*	N/A	Rençber et al., 2016
	Solid Lipid Nanoparticles	N/A	N/A	*Candida albicans, Candida glabrata, Candida parapsilosis*	Moazeni et al., 2016
		Topical	*Candida albicans*	N/A	Gupta and Vyas, 2012
Itraconazole	Lipid nanoparticles	Inhalation	*Aspergillus fumigatus*	N/A	Pardeike et al., 2016
	Polymeric nanoparticles	N/A	*Candida albicans*	N/A	Qiu et al., 2015
		N/A	N/A	*Aspergillus flavus*	Patel et al., 2011
		N/A	N/A	*Candida albicans, Aspergillus fumigatus*	Essa et al., 2012
		N/A	N/A	*Paracoccidioides brasiliensis*	Cunha-Azevedo et al., 2011
		N/A	N/A	*Aspergillus flavus*	Patel et al., 2010
	Solid Lipid Nanoparticles	Ocular	N/A	*Aspergillus flavus*	Mohanty et al., 2015
Miconazole	Liposomes	Topical	*Candida albicans*	N/A	Pandit et al., 2014
	Solid Lipid Nanoparticles	Topical	*Candida albicans*	N/A	Jain et al., 2010
Nystatin	Polymeric nanoparticles	N/A	N/A	*Candida albicans*	Mohammadi et al., 2017
Voriconazole	Polymeric nanoparticles	Oral	*Candida albicans*	N/A	Peng et al., 2008

## Nanopreparations for polyenes

Among antifungal drugs, the investigation surrounding nanostructured delivery systems for polyenes are the most reported, the majority of them concerning Amphotericin B delivery. This is not surprising, since Amphotericin B is still the gold standard for antifungal therapy of severe systemic mycosis and the drug with the most potent antifungal capacity (Arnold et al., 2010). Amphotericin B nanoparticles have been investigated since 1980s, providing the opportunity to circumvent infusion-related side effects and nephrotoxicity, which are the main limiting factors concerning therapy with this drug. Further, because of the Amphotericin B poor solubility and oral bioavailability (Kang et al., 2010), such formulations are also intended to improve these characteristics, making it suitable for oral delivery.

Currently, lipid formulations for Amphotericin B are commercially available (Walsh et al., 1999; Kleinberg, 2006). The most successful is Ambisome®, which is considered a truly liposomal formulation, where Amphotericin B is embedded in the unilamellar liposome bilayer of about 45–80 nm (Adler-Moore, 1994). One of the advantages brought by this formulation is that it causes less toxicity and remains at high peaks in the circulation, presenting in a high biodistribution. A study using the murine model of pulmonary aspergillosis showed that Ambisome® was able to increase the survival of animals without toxicity, allowing the administration of a 10-fold higher dose than in animals receiving the conventional formulation in sodium deoxycholate (Takemoto, 2006). Liposomal formulations for topical applications have also been explored (Kang et al., 2010; Perez et al., 2016). Some of these preparations aim to increase the permanency of the formulation at the site of action, releasing the drug for longer time. Unfortunately, together with the higher biosafety, it was reported that antifungal effectiveness of Amphotericin B lipid formulations may be reduced in comparison to that of free drug (Andes et al., 2006; Burgess et al., 2013). In addition to that, the high costs are among the limitations to their widespread use (Dismukes, 2000; Falci et al., 2011; Italia et al., 2011; Van de Ven et al., 2012), encouraging the search for new options for Amphotericin B delivery.

In order to diminish costs of manufacturing, Nanosomal Amphotericin B was developed using phosphatidylcholine and sodium cholesteryl sulfate as excipients avoiding the use of organic solvents or detergents in the preparation (Sheikh et al., 2010). Nanosomal Amphotericin B induced less lysis of red blood cells than Amphotericin B sodium deoxycholate and was comparable to Ambisome®. No symptoms of toxicity, mortality or significant body weight reduction were observed in rabbits daily treated with this formulation for 28 days, with no hematological and gross pathological abnormalities. Besides, in mice disseminated *A. fumigatus* infection, intravenous administration of Nanosomal Amphotericin B resulted in 90% survival while only 30% survival with Ambisome®.

Anionic and PEG lipid nanoparticles were developed by Jung et al. (2009) for intravenous delivery of Amphotericin B (Jung et al., 2009). This formulation presented lower cytotoxicity against human kidney cells than Fungizone® and Ambisome®, and much lower hematotoxicity than that of Fungizone®. Antifungal activity *in vitro* of Amphotericin B lipid nanoparticles against *C. albicans* and *A. fumigatus* was better than the commercialized formulations and *in vivo* administration for the treatment of murine systemic aspergilloma was more effective than that of Ambisome®.

Burgess et al. (2013) developed a formulation consisting of a protein-phospholipid bioparticle (NanoDisk) containing a “super aggregate” form of amphotericin B (ND–AMB) (Burgess et al., 2013). ND–AMB presented lower *C. albicans* and *A. fumigatus* minimum inhibitory concentrations than those observed for Ambisome®. It also induced no kidney or liver toxicity in mice. In addition, in *C. albicans* infected immune-competent mice, ND-AMB treatment was as effective as sodium deoxycholate amphotericin B or Ambisome®, whereas in a leukopenic model of candidiasis, the 50% effective dose of ND-AMB was around threefold lower than Ambisome®.

In addition to the lipid formulations, polymeric systems for the delivery of Amphotericin B have been developed by several research groups (Table 2). The results proved this type of preparation to be satisfactory for the efficient delivery of this drug by several routes, such as intravenously (Amaral et al., 2009; Tang et al., 2014; Souza et al., 2015) and orally (Italia et al., 2009, 2011, 2012). The biological response may vary according to the type of polymer used or even by combining different polymer types. An example is the formulation developed by Tang et al. (2014), in which AMB was encapsulated within PLGA in association with ε-caprolactone. In this combination, the authors reported an AMB encapsulation efficiency of 84% and the same fungicidal effect on *C. albicans* as for the free drug (Tang et al., 2014). The proposed formulation was less toxic and caused lower mortality than free AMB, proving, as noted before, the potential of polymeric nanoparticles to protect against cytotoxicity but preserving the same fungicidal efficacy (Amaral et al., 2009).

Amaral et al. (2009) developed a nanostructured formulation for Amphotericin B within PLGA functionalized with dimercaptosuccinic acid (DMSA). This acid presents tropism to the lungs, being suitable to be incorporation in formulations for drug delivery to this organ (Amaral et al., 2009, 2010). In this study, authors noted that intraperitoneal administration of the formulation was able to cause the same therapeutic effect in murine model of paracoccidioidomycosis compared to sodium deoxycholate Amphotericin B. However, the great advantage of this polymeric formulation was that it was given to the animals every 3 days, considering the slow release of Amphotericin B from nanoparticles, in contrast to the conventional formulation, which was administrated daily. Complementary studies conducted by the same research group showed that amphotericin B PLGA nanoparticles were also comparable to Ambisome® in the therapy of murine paracoccidioidomycosis (Souza et al., 2015). In addition, both studies reported no undesirable side effects in animals at the dose used. High concentrations of Amphotericin B were found in lungs, liver and spleen of animals treated with this polymeric formulation (Souza et al., 2015).

Van de Ven et al. (2012) showed that both Amphotericin B PLGA nanoparticles and nanoemulsion had *in vitro* antifungal capacity against *C. albicans, A. fumigatus*, and *Trichophyton rubrum* and were less hemolytic than sodium deoxycholate Amphotericin B (Van de Ven et al., 2012). In addition, both formulations were effective against murine disseminated aspergillosis after intraperitoneal administration, with similar or even better antifungal capacity than Fungizone® and Ambisome® at equivalent doses. Italia et al. (2011) proposed Amphotericin B PLGA nanoparticles for oral delivery, which significantly reduced fungal burden during invasive pulmonary and disseminated aspergillosis (Italia et al., 2011).

In a very interesting approach, Tang et al. (2015a) developed specialized pH-responsive Amphotericin B PLGA nanoparticles conjugated with poly(L-histidine) and PEG that had high affinity for fungal cell wall elements under acidic conditions and were further modified for increased targeting efficacy with anti-*C. albicans* antibody (Tang et al., 2015a). The formulation had reduced hemolytic activity and cytotoxicity on human renal tubular epithelial cells, and was effective against *C. albicans* both *in vivo* and *in vitro* after intravenous administration. Nystatin loaded PLGA nanoparticles functionalized with Glucosamine were formulated by Mohammadi et al. (2017) to enhance the adhesion of nanoparticles to *C. albicans* cell walls (Mohammadi et al., 2017). The nanoparticles exhibited higher antifungal activity than free Nystatin, suggesting an increase in Nystatin levels in fungal cell membrane by entrapment in the polymeric matrix.

Chitosan is a natural polysaccharide with cationic nature and mucoadhesive proprieties that has been frequently used to build biodegradable polymeric nanoparticles for drug delivery. Amphotericin B chitosan nanoparticles were developed by Serrano et al. (2015) intending to avoid drug gastrointestinal degradation, improve stability and enhance bioavailability in target organs such as lung, liver, and spleen, while diminishing kidney exposure (Serrano et al., 2015). They have demonstrated that oral administration of these nanoparticles was effective in the treatment of murine models of visceral leishmaniasis, candidiasis and aspergillosis, having comparable efficacy to parenteral Ambisome®. Chitosan nanoparticles were used by Sanchez et al. (2014) for topical delivery of Amphotericin B to burn wounds infected with *C. albicans*, with enhanced tissue healing and even better antifungal activity than sodium deoxycholate formulation (Sanchez et al., 2014). Amphotericin B loaded nanoparticles made of lecithin and chitosan were proposed by Chhonker et al. (2015) for prolonged ocular application such as during fungal keratitis (Chhonker et al., 2015). The formulation presented *in vitro* antifungal efficacy against *C. albicans* and *A. fumigatus*, pronounced mucoadhesive properties and improved bioavailablity as compared with Fungizone®.

Approaches to increase Amphotericin B delivery into central nervous system (CNS) are also under investigation. Amphotericin B polybutylcyanoacrylate nanoparticles modified with polysorbate 80 were used as therapy for cryptococcal meningitis murine model (Xu et al., 2011). Nanoparticles were detected in the brain 30 min after systemic administration into mice with a higher concentration than Amphotericin B liposomes. Survival rate of mice treated with nanoparticles was significantly higher than that of sodium deoxycholate and liposomal amphotericin B treated groups, with lower fungal burden in brain tissue. Another formulation based in transferrin transcytosis at the blood-brain barrier was proposed by Tang et al. (2015c). In this study, anti-transferrin receptor antibody-modified Amphotericin B-loaded PLA-PEG nanoparticles were developed and presented significant reduction of CNS fungal burden and increased mouse survival time when administered intravenously for the treatment of meningitis induced by *C. glabrata* inoculation (Tang et al., 2015c).

Shirkhani et al. (2015) developed Amphotericin B polymethacrylic acid nanoparticles for the prophylaxis of *A. fumigatus* infection in a transplant immunosuppression murine model with invasive aspergillosis. The formulation was given by nebulization and prevented fungal growth and lung inflammation (Shirkhani et al., 2015).

Amphotericin B magnetic nanoparticles were reported by Saldanha et al. (2016). In this study, authors developed Amphotericin B loaded lauric acid pre-coated magnetite nanoparticles and showed nanocomplex antifungal activity both *in vivo* and i*n vitro* against *P. brasiliensis* infection (Saldanha et al., 2016). The nanocomplex was more cytotoxic to fungal cells than to human urinary cells and murine peritoneal macrophages *in vitro*, while no biochemical and histopathological alterations were observed during intranasal therapy against murine paracoccidioidomycosis. The formulation exhibited similar antifungal activity to that of sodium deoxycholate Amphotericin B administrated intraperitonealy, with the advantage to allow a three-fold reduction in the number of applications and to be suitable for nasal delivery. Another Amphotericin B magnetic liposomal system were proposed by Zhao et al. (2014) aiming to enhance drug selectivity to CNS during fungal infections. The liposomal system was administered via carotid artery route in SD rats and could improve drug concentration and enhance magnetic targeting to brain tissue in the presence of a magnetic field. Amphotericin B and nystatin magnetic nanoparticles were also designed by Niemirowicz et al. (2016). Both nanosystems displayed stronger fungicidal activity than unbound drugs against *Candida* spp. (Niemirowicz et al., 2016). Nanosystems were more potent than free agents when tested against *Candida* strains and were able to prevent *Candida* biofilm formation more effectively with lower hemolytic capacity.

Some fungal pathogens have the ability to reside inside host cells, such as macrophages. Therefore, nanoparticles uptake by host cells is very desirable as a strategy to enhance antifungal abilities against intracellular fungi. Zia et al. (2015) showed that killing of intracellular *C. albicans* after treatment with amphotericin B polyglutamic acid nanoparticles was higher than with Ambisome® (Zia et al., 2015). Amphotericin B is also used for the treatment of infections caused by *Leishmania* spp., which is an intracellular parasite, so that intense investigation has been done in order to enhance drug antiparasitic effects throughout nanostruturation (Asthana et al., 2015; Jain et al., 2015; Bose et al., 2016).

## Nanopreparations for azoles

The increase of oral bioavailability of azoles is one of the advantages conferred by the use of nanotechnology for this class of drug. It is interesting to note that although preparing a formulation at the nanoscale may suggest similar results when using the same type of nanoparticle and material, this is not always true. For example, a study encapsulating econazole and clotrimazole in both PLGA and alginate (Pandey et al., 2005) demonstrated that the latter seems to be better at improving pharmacokinetic parameters. Though, both proved to be effective for the oral route.

Considering the severity of some fungal infections affecting the lungs, nanostructured delivery systems for drugs capable of reaching this organ are sought. Some studies showed the tropism for the lungs by PLGA nanoparticles. The PLGA-encapsulated voriconazole showed a higher accumulation in the lungs when compared with free voriconazole (Das et al., 2015). This characteristic was also observed by other researchers when they also encapsulated voriconazole in PLGA for nasal administration. The study demonstrated the system allows the early release of the drug in the lungs in the first 2 h, followed by a sustained release during 15 days (Sinha et al., 2013). Itraconazole PLGA nanoparticles conjugated with dimercaptosuccinic acid were developed for pulmonary delivery and induced antifungal inhibition against *P. brasiliensis* with lower *in vitro* cytotoxicity than free drug (Cunha-Azevedo et al., 2011).

Voriconazole loaded PLGA nanoparticles were developed by Peng et al. (2008) in order to improve drug bioavailability and stability for oral delivery (Peng et al., 2008). Nanoparticles had a more persistent and potent antifungal effect than free voriconazole both *in vitro* and *in vivo* in a systemic candidiasis murine model.

Other triazole loaded polymeric nanostructured formulations have been investigated and had *in vitro* antifungal activities against *A. flavus* (Patel et al., 2010, 2011), *A. fumigatus* (Essa et al., 2012), and *C. albicans* (Qiu et al., 2015). Some polymers have mucoadhesive properties, such as chitosan, and can be used to prepare drug delivery systems for mucosa from the eyes, mouth and vagina. Rençber et al. (2016) developed a buccal mucoadhesive nanoparticle containing fluconazole and EUDRAGIT®, a nonbiodegradable and cationic copolymer, for the local treatment of oral candidiasis. The formulation presented *in vitro* antifungal activity against *C. albicans* for an extended period, and no cytotoxic effect in chinese hamster ovary cells was observed. Rabbits with oral candidiasis were successfully treated with local administration of the nanoparticles once a day (Rençber et al., 2016). More sophisticated drug delivery systems formed by different types of nanoparticles can be prepared (Jøraholmen et al., 2014). For example, in one polymer the drug is encapsulated and this polymer is coated with another to present the proper *in vivo* application.

Ultraflexible liposomes carrying miconazole also showed to be more efficient and able to penetrate the cell barrier carrying the drug. As they are able to adapt their shape according to the characteristics of the near microenvironment, they are able to penetrate and release the drug more efficiently (Pandit et al., 2014). It is also possible to promote the release of miconazole only to the skin, without penetrating it or reaching the epidermis (Elmoslemany et al., 2012), which is important for reducing toxicity.

Solid lipid nanoparticles (SLN) have been intensively investigated for topical delivery of azole antifungals. Jain et al. (2010) developed a formulation of miconazole nitrate loaded SLN-bearing hydrogel for skin delivery (Jain et al., 2010). Studies indicated that miconazole SLN-bearing hydrogel resulted in considerably less skin irritation as compared to miconazole hydrogel and suspension after 24 h of application. In addition, micozanole SLN treatment of cutaneous candidiasis in albino rats presented greater efficiency and fast recovery. Mohanty et al. (2015) investigated SLNs for topical ocular delivery of itraconazole (Mohanty et al., 2015). Permeation of itraconazole in freshly excised goat corneas was observed, and SLN inhibited *Aspergillus flavus in vitro* growth, indicating antimicrobial efficacy of formulations.

Moazeni et al. (2016) tested fluconazole-loaded SLN efficacy against fluconazole-resistant *Candida* spp. strains. Fluconazole-resistant *C. albicans, C. parapsilosis*, and *C. glabrata* strains behaved as susceptible strains after treatment with fluconazole SLN, emphasizing the promising benefits of nanostructuration for the delivery of antifungal agents.

## Concluding remarks

The high incidence of fungal infections is a problem that can be aggravated mainly by the increase of the elderly population and also by immunocompromised patients. In this way, it is important that new and more effective therapies are developed for the treatment of these mycoses. One strategy that has received importance for this purpose is the development of drugs applying the principles of nanotechnology. It is possible to use the same conventional drugs, but aiming to increase its therapeutic efficacy and also the reduction of its side effects. Thus, unlike the basic development of a novel antifungal molecule, which is also of relevant importance, conventional formulations may be improved in effectiveness by the resources of Nanobiotechnology.

## Author contributions

All authors listed, have made substantial, direct and intellectual contribution to the work, and approved it for publication.

## Funding

Ana Camila Oliveira Souza is funded by grant 2015/10390-6 from the Fundação de Amparo à Pesquisa de São Paulo (FAPESP).

### Conflict of interest statement

The authors declare that the research was conducted in the absence of any commercial or financial relationships that could be construed as a potential conflict of interest.

## References

[B1] Adler-Moore J. (1994). AmBisome targeting to fungal infections. Bone Marrow Transplant. 14(Suppl. 5), S3–S7. 7703928

[B2] Adler-Moore J. P. Gangneux J. P. Pappas P. G. (2016). Comparison between liposomal formulations of amphotericin B. Med. Mycol. 54, 223–231. 10.1093/mmy/myv111 26768369

[B3] Agger E. M. Rosenkrands I. Hansen J. Brahimi K. Vandahl B. S. Aagaard C.. (2008). Cationic liposomes formulated with synthetic mycobacterial cordfactor (CAF01): a versatile adjuvant for vaccines with different immunological requirements. PLoS ONE 3:e3116. 10.1371/journal.pone.0003116 18776936 PMC2525815

[B4] Albasarah Y. Y. Somavarapu S. Stapleton P. Taylor K. M. (2010). Chitosan-coated antifungal formulations for nebulisation. J. Pharm. Pharmacol. 62, 821–828. 10.1211/jpp.62.05.0002 20636869

[B5] Amaral A. C. Bocca A. L. Ribeiro A. M. Nunes J. Peixoto D. L. Simioni A. R.. (2009). Amphotericin B in poly(lactic-co-glycolic acid) (PLGA) and dimercaptosuccinic acid (DMSA) nanoparticles against paracoccidioidomycosis. J. Antimicrob. Chemother. 63, 526–533. 10.1093/jac/dkn539 19151037

[B6] Amaral A. C. Felipe M. S. S. (2013). Nanobiotechnology: an efficient approach to drug delivery of unstable biomolecules. Curr. Protein Pept. Sci. 14, 588–594. 10.2174/1389203711209070632 23968343

[B7] Amaral A. C. Marques A. F. Muñoz J. E. Bocca A. L. Simioni A. R. Tedesco A. C.. (2010). Poly(lactic acid-glycolic acid) nanoparticles markedly improve immunological protection provided by peptide P10 against murine paracoccidioidomycosis. Br. J. Pharmacol. 159, 1126–1132. 10.1111/j.1476-5381.2009.00617.x 20136827 PMC2839270

[B8] Andes D. Safdar N. Marchillo K. Conklin R. (2006). Pharmacokinetic-pharmacodynamic comparison of amphotericin B (AMB) and two lipid-associated AMB preparations, liposomal AMB and AMB lipid complex, in murine candidiasis models. Antimicrob. Agents Chemother. 50, 674–684. 10.1128/AAC.50.2.674-684.2006 16436726 PMC1366906

[B9] Arnold T. M. Dotson E. Sarosi G. A. Hage C. A. (2010). Traditional and emerging antifungal therapies. Proc. Am. Thorac. Soc. 7, 222–228. 10.1513/pats.200906-048AL 20463252

[B10] Asthana S. Gupta P. K. Jaiswal A. K. Dube A. Chourasia M. K. (2015). Overexpressed macrophage mannose receptor targeted nanocapsules- mediated cargo delivery approach for eradication of resident parasite: *in vitro* and *in vivo* studies. Pharm. Res. 32, 2663–2677. 10.1007/s11095-015-1651-0 25715698

[B11] Bartner E. Zinnes H. Moe R. A. Kuleska J. S. (1958). Studies on a new solubilized preparation of amphotericin B. Antibiot. Annu. 5, 53–58. 13521782

[B12] Bhowmick S. Ravindran R. Ali N. (2008). gp63 in stable cationic liposomes confers sustained vaccine immunity to susceptible BALB/c mice infected with *Leishmania donovani*. Infect. Immun. 76, 1003–1015. 10.1128/IAI.00611-07 18195029 PMC2258822

[B13] Bolhassani A. Javanzad S. Saleh T. Hashemi M. Aghasadeghi M. R. Sadat S. M. (2014). Polymeric nanoparticles. Hum. Vaccin. Immunother. 10, 321–332. 10.4161/hv.26796 24128651 PMC4185908

[B14] Bonifaz A. Vázquez-González D. Perusquía-Ortiz A. M. (2011). Endemic systemic mycoses: coccidioidomycosis, histoplasmosis, paracoccidioidomycosis and blastomycosis. J. Dtsch. Dermatol. Ges. 9, 705–715. 10.1111/j.1610-0387.2011.07731.x 21722309

[B15] Borborema S. E. Schwendener R. A. Osso J. A. de Andrade H. F. Jr. do Nascimento N. (2011). Uptake and antileishmanial activity of meglumine antimoniate-containing liposomes in Leishmania (Leishmania) major-infected macrophages. Int. J. Antimicrob. Agents 38, 341–347. 10.1016/j.ijantimicag.2011.05.012 21783345

[B16] Bose P. P. Kumar P. Dwivedi M. K. (2016). Hemoglobin guided nanocarrier for specific delivery of amphotericin B to Leishmania infected macrophage. Acta Trop. 158, 148–159. 10.1016/j.actatropica.2016.02.026 26945483

[B17] Bozzuto G. Molinari A. (2015). Liposomes as nanomedical devices. Int. J. Nanomedicine 10, 975–999. 10.2147/IJN.S68861 25678787 PMC4324542

[B18] Brown G. D. Denning D. W. Gow N. A. Levitz S. M. Netea M. G. White T. C. (2012a). Hidden killers: human fungal infections. Sci. Transl. Med. 4, 165rv13. 10.1126/scitranslmed.3004404 23253612

[B19] Brown G. D. Denning D. W. Levitz S. M. (2012b). Tackling human fungal infections. Science 336, 647–647. 10.1126/science.1222236 22582229

[B20] Burgess B. L. He Y. Baker M. M. Luo B. Carroll S. F. Forte T. M.. (2013). NanoDisk containing super aggregated amphotericin B: a high therapeutic index antifungal formulation with enhanced potency. Int. J. Nanomedicine 8, 4733–4743. 10.2147/IJN.S50113 24379661 PMC3867322

[B21] Butts A. Krysan D. J. (2012). Antifungal drug discovery: something old and something new. PLoS Pathog. 8:e1002870. 10.1371/journal.ppat.1002870 22969422 PMC3435257

[B22] Caffrey A. K. Obar J. J. (2016). Alarmin(g) the innate immune system to invasive fungal infections. Curr. Opin. Microbiol. 32, 135–143. 10.1016/j.mib.2016.06.002 27351354 PMC4983492

[B23] Carrillo-Muñoz A. J. Giusiano G. Ezkurra P. A. Quindós G. (2006). Antifungal agents: mode of action in yeast cells. Rev. Esp. Quimioter. 19, 130–139. 16964330

[B24] Casa D. M. Carraro T. C. de Camargo L. E. Dalmolin L. F. Khalil N. M. Mainardes R. M. (2015). Poly(L-lactide) nanoparticles reduce amphotericin B cytotoxicity and maintain its *in vitro* antifungal activity. J. Nanosci. Nanotechnol. 15, 848–854. 10.1166/jnn.2015.9177 26328449

[B25] Chaudhari M. B. Desai P. P. Patel P. A. Patravale V. B. (2016). Solid lipid nanoparticles of amphotericin B (AmbiOnp): *in vitro* and *in vivo* assessment towards safe and effective oral treatment module. Drug Deliv. Transl. Res. 6, 354–364. 10.1007/s13346-015-0267-6 26712123

[B26] Chhonker Y. S. Prasad Y. D. Chandasana H. Vishvkarma A. Mitra K. Shukla P. K.. (2015). Amphotericin-B entrapped lecithin/chitosan nanoparticles for prolonged ocular application. Int. J. Biol. Macromol. 72, 1451–1458. 10.1016/j.ijbiomac.2014.10.014 25453292

[B27] Como J. A. Dismukes W. E. (1994). Oral azole drugs as systemic antifungal therapy. N. Engl. J. Med. 330, 263–272. 10.1056/NEJM199401273300407 8272088

[B28] Cunha-Azevedo E. P. Silva J. R. Martins O. P. Siqueira-Moura M. P. Bocca A. L. Felipe M. S.. (2011). *In vitro* antifungal activity and toxicity of itraconazole in DMSA-PLGA nanoparticles. J. Nanosci. Nanotechnol. 11, 2308–2314. 10.1166/jnn.2011.3576 21449386

[B29] Das P. J. Paul P. Mukherjee B. Mazumder B. Mondal L. Baishya R.. (2015). Pulmonary delivery of voriconazole loaded nanoparticles providing a prolonged drug level in lungs: a promise for treating fungal infection. Mol. Pharm. 12, 2651–2664. 10.1021/acs.molpharmaceut.5b00064 25941882

[B30] Das S. Suresh P. K. (2011). Nanosuspension: a new vehicle for the improvement of the delivery of drugs to the ocular surface. Application to amphotericin B. Nanomedicine 7, 242–247. 10.1016/j.nano.2010.07.003 20692375

[B31] Denning D. W. Hope W. W. (2010). Therapy for fungal diseases: opportunities and priorities. Trends Microbiol. 18, 195–204. 10.1016/j.tim.2010.02.004 20207544

[B32] de Sá F. A. Taveira S. F. Gelfuso G. M. Lima E. M. Gratieri T. (2015). Liposomal voriconazole (VOR) formulation for improved ocular delivery. Colloids Surf. B Biointerfaces 133, 331–338. 10.1016/j.colsurfb.2015.06.036 26123854

[B33] Dismukes W. E. (2000). Introduction to antifungal drugs. Clin. Infect. Dis. 30, 653–657. 10.1086/313748 10770726

[B34] Dube A. Reynolds J. L. Law W.-C. Maponga C. C. Prasad P. N. Morse G. D. (2014). Multimodal nanoparticles that provide immunomodulation and intracellular drug delivery for infectious diseases. Nanomedicine 10, 831–838. 10.1016/j.nano.2013.11.012 24333593

[B35] Dupont B. (2002). Overview of the lipid formulations of amphotericin B. J. Antimicrob. Chemother. 49(Suppl. 1), 31–36. 10.1093/jac/49.suppl_1.31 11801578

[B36] Elmoslemany R. M. Abdallah O. Y. El-Khordagui L. K. Khalafallah N. M. (2012). Propylene glycol liposomes as a topical delivery system for miconazole nitrate: comparison with conventional liposomes. AAPS PharmSciTech 13, 723–731. 10.1208/s12249-012-9783-6 22566173 PMC3364396

[B37] Essa S. Louhichi F. Raymond M. Hildgen P. (2012). Improved antifungal activity of itraconazole-loaded PEG/PLA nanoparticles. J. Microencapsul. 30, 1–13. 10.3109/02652048.2012.714410 22894166

[B38] Falci D. R. dos Santos R. P. Wirth F. Goldani L. Z. (2011). Continuous infusion of amphotericin B deoxycholate: an innovative, low-cost strategy in antifungal treatment. Mycoses 54, 91–98. 10.1111/j.1439-0507.2009.01805.x 19878457

[B39] Finkelstein A. Holz R. (1973). Aqueous pores created in thin lipid membranes by the polyene antibiotics nystatin and amphotericin B. Membranes (Basel). 2, 377–408. 4585230

[B40] Francis P. Walsh T. J. (1992). Evolving role of flucytosine in immunocompromised patients: new insights into safety, pharmacokinetics, and antifungal therapy. Clin. Infect. Dis. 15, 1003–1018. 1457631 10.1093/clind/15.6.1003

[B41] Gangadhar K. N. Adhikari K. Srichana T. (2014). Synthesis and evaluation of sodium deoxycholate sulfate as a lipid drug carrier to enhance the solubility, stability and safety of an amphotericin B inhalation formulation. Int. J. Pharm. 471, 430–438. 10.1016/j.ijpharm.2014.05.066 24907597

[B42] Georgopapadakou N. H. (1998). Antifungals: mechanism of action and resistance, established and novel drugs. Curr. Opin. Microbiol. 1, 547–557. 10.1016/S1369-5274(98)80087-8 10066533

[B43] Georgopapadakou N. H. (2001). Update on antifungals targeted to the cell wall: focus on β-1,3-glucan synthase inhibitors. Expert Opin. Investig. Drugs 10, 269–280. 10.1517/13543784.10.2.269 11178340

[B44] Goughenour K. D. Rappleye C. A. (2017). Antifungal therapeutics for dimorphic fungal pathogens. Virulence 8, 211–221. 10.1080/21505594.2016.1235653 27646561 PMC5354166

[B45] Groll A. H. Gea-Banacloche J. C. Glasmacher A. Just-Nuebling G. Maschmeyer G. Walsh T. J. (2003). Clinical pharmacology of antifungal compounds. Infect. Dis. Clin. North Am. 17, 159–191. 10.1016/S0891-5520(02)00068-5 12751265

[B46] Groll A. H. Piscitelli S. C. Walsh T. J. (1998). Clinical pharmacology of systemic antifungal agents: a comprehensive review of agents in clinical use, current investigational compounds, and putative targets for antifungal drug development. Adv. Pharmacol. 44, 343–500. 10.1016/S1054-3589(08)60129-5 9547888

[B47] Gulati M. Bajad S. Singh S. Ferdous A. J. Singh M. (1998). Development of liposomal amphotericin B formulation. J. Microencapsul. 15, 137–151. 10.3109/02652049809006844 9532520

[B48] Gupta M. Vyas S. P. (2012). Development, characterization and *in vivo* assessment of effective lipidic nanoparticles for dermal delivery of fluconazole against cutaneous candidiasis. Chem. Phys. Lipids 165, 454–461. 10.1016/j.chemphyslip.2012.01.006 22309657

[B49] Han K. Miah M. A. J. Shanmugam S. Yong C. S. Choi H.-G. Kim J. A.. (2007). Mixed micellar nanoparticle of amphotericin B and poly styrene-block-poly ethylene oxide reduces nephrotoxicity but retains antifungal activity. Arch. Pharm. Res. 30, 1344–1349. 10.1007/BF02980276 18038914

[B50] Havlickova B. Czaika V. A. Friedrich M. (2008). Epidemiological trends in skin mycoses worldwide. Mycoses 51, 2–15. 10.1111/j.1439-0507.2008.01606.x 18783559

[B51] Heitman J. (2011). Microbial pathogens in the fungal kingdom. Fungal Biol. Rev. 25, 48–60. 10.1016/j.fbr.2011.01.003 21528015 PMC3081590

[B52] Hsuchen C. C. Feingold D. S. (1973). Selective membrane toxicity of the polyene antibiotics: studies on natural membranes. Antimicrob. Agents Chemother. 4, 316–319. 4586145 10.1128/aac.4.3.316 PMC444549

[B53] Hussain A. Samad A. Singh S. K. Ahsan M. N. Haque M. W. Faruk A.. (2016). Nanoemulsion gel-based topical delivery of an antifungal drug: *in vitro* activity and *in vivo* evaluation. Drug Deliv. 23, 642–647. 10.3109/10717544.2014.933284 25013957

[B54] Hussein M. Z. Al Ali S. Geilich B. M. El Zowalaty M. E. Webster T. J. (2014). Synthesis, characterization, and antimicrobial activity of an ampicillin-conjugated magnetic nanoantibiotic for medical applications. Int. J. Nanomedicine 9, 3801–3814. 10.2147/IJN.S61143 25143729 PMC4134181

[B55] Italia J. L. Kumar M. N. V. R. Carter K. C. (2012). Evaluating the potential of polyester nanoparticles for per oral delivery of amphotericin B in treating visceral leishmaniasis. J. Biomed. Nanotechnol. 8, 695–702. 10.1166/jbn.2012.1414 22852479

[B56] Italia J. L. Sharp A. Carter K. C. Warn P. Kumar M. N. (2011). Peroral amphotericin B polymer nanoparticles lead to comparable or superior *in vivo* antifungal activity to that of intravenous Ambisome® or Fungizone™. PLoS ONE 6:e25744. 10.1371/journal.pone.0025744 21998690 PMC3188565

[B57] Italia J. L. Yahya M. M. Singh D. Ravi Kumar M. N. (2009). Biodegradable nanoparticles improve oral bioavailability of amphotericin B and show reduced nephrotoxicity compared to intravenous fungizone®. Pharm. Res. 26, 1324–1331. 10.1007/s11095-009-9841-2 19214716

[B58] Jain K. Verma A. K. Mishra P. R. Jain N. K. (2015). Characterization and evaluation of amphotericin B loaded MDP conjugated poly(propylene imine) dendrimers. Nanomedicine 11, 705–713. 10.1016/j.nano.2014.11.008 25596078

[B59] Jain S. Jain S. Khare P. Gulbake A. Bansal D. Jain S. K. (2010). Design and development of solid lipid nanoparticles for topical delivery of an anti-fungal agent. Drug Deliv. 17, 443–451. 10.3109/10717544.2010.483252 20486871

[B60] Jain T. K. Richey J. Strand M. Leslie-Pelecky D. L. Flask C. A. Labhasetwar V. (2008). Magnetic nanoparticles with dual functional properties: drug delivery and magnetic resonance imaging. Biomaterials 29, 4012–4021. 10.1016/j.biomaterials.2008.07.004 18649936 PMC2593647

[B61] Jiang L. Li L. He X. Yi Q. He B. Cao J.. (2015). Overcoming drug-resistant lung cancer by paclitaxel loaded dual-functional liposomes with mitochondria targeting and pH-response. Biomaterials 52, 126–139. 10.1016/j.biomaterials.2015.02.004 25818419

[B62] Jøraholmen M. W. Vanić Z. Tho I. Skalko-Basnet N. (2014). Chitosan-coated liposomes for topical vaginal therapy: assuring localized drug effect. Int. J. Pharm. 472, 94–101. 10.1016/j.ijpharm.2014.06.016 24928137

[B63] Jung S. H. Lim D. H. Jung S. H. Lee J. E. Jeong K.-S. Seong H.. (2009). Amphotericin B-entrapping lipid nanoparticles and their *in vitro* and *in vivo* characteristics. Eur. J. Pharm. Sci. 37, 313–320. 10.1016/j.ejps.2009.02.021 19491021

[B64] Kang J.-W. Davaa E. Kim Y.-T. Park J.-S. (2010). A new vaginal delivery system of amphotericin B: a dispersion of cationic liposomes in a thermosensitive gel. J. Drug Target. 18, 637–644. 10.3109/10611861003649712 20192816

[B65] Kleinberg M. (2006). What is the current and future status of conventional amphotericin B? Int. J. Antimicrob. Agents 27(Suppl. 1), 12–16. 10.1016/j.ijantimicag.2006.03.013 16707251

[B66] Kumar A. Jena P. K. Behera S. Lockey R. F. Mohapatra S. Mohapatra S. (2010). Multifunctional magnetic nanoparticles for targeted delivery. Nanomedicine 6, 64–69. 10.1016/j.nano.2009.04.002 19446653 PMC3319306

[B67] Kumar R. Sinha V. R. (2016). Solid lipid nanoparticle: an efficient carrier for improved ocular permeation of voriconazole. Drug Dev. Ind. Pharm. 42, 1956–1967. 10.1080/03639045.2016.1185437 27143048

[B68] Laniado-Laborín R. Cabrales-Vargas M. N. (2009). Amphotericin B: side effects and toxicity. Rev. Iberoam. Micol. 26, 223–227. 10.1016/j.riam.2009.06.003 19836985

[B69] Malathi S. Balasubramanian S. (2011). Synthesis of biodegradable polymeric nanoparticles and their controlled drug delivery for tuberculosis. J. Biomed. Nanotechnol. 7, 150–151. 10.1166/jbn.2011.1244 21485846

[B70] McCormack P. L. (2015). Isavuconazonium: first global approval. Drugs 75, 817–822. 10.1007/s40265-015-0398-6 25902926

[B71] Mesa-Arango A. C. Scorzoni L. Zaragoza O. (2012). It only takes one to do many jobs: amphotericin B as antifungal and immunomodulatory drug. Front. Microbiol. 3:286. 10.3389/fmicb.2012.00286 23024638 PMC3441194

[B72] Moazeni M. Kelidari H. R. Saeedi M. Morteza-Semnani K. Nabili M. Gohar A. A.. (2016). Time to overcome fluconazole resistant Candida isolates: solid lipid nanoparticles as a novel antifungal drug delivery system. Colloids Surf. B Biointerfaces 142, 400–407. 10.1016/j.colsurfb.2016.03.013 26974361

[B73] Mohammadi G. Shakeri A. Fattahi A. Mohammadi P. Mikaeili A. Aliabadi A.. (2017). Preparation, physicochemical characterization and anti-fungal evaluation of nystatin-loaded PLGA-glucosamine nanoparticles. Pharm. Res. 34, 301–309. 10.1007/s11095-016-2062-6 27928646

[B74] Mohanty B. Majumdar D. K. Mishra S. K. Panda A. K. Patnaik S. (2015). Development and characterization of itraconazole-loaded solid lipid nanoparticles for ocular delivery. Pharm. Dev. Technol. 20, 458–464. 10.3109/10837450.2014.882935 24490828

[B75] Moritz M. Geszke-Moritz M. (2015). Recent developments in application of polymeric nanoparticles as drug carriers. Adv. Clin. Exp. Med. 24, 749–758. 10.17219/acem/31802 26768624

[B76] Mukherjee P. K. Sheehan D. J. Hitchcock C. A. Ghannoum M. A. (2005). Combination treatment of invasive fungal infections. Clin. Microbiol. Rev. 18, 163–194. 10.1128/CMR.18.1.163-194.2005 15653825 PMC544182

[B77] Nett J. E. Andes D. R. (2016). Antifungal agents: spectrum of activity, pharmacology, and clinical indications. Infect. Dis. Clin. North Am. 30, 51–83. 10.1016/j.idc.2015.10.012 26739608

[B78] Niemirowicz K. Durnaś B. Tokajuk G. Głuszek K. Wilczewska A. Z. Misztalewska I.. (2016). Magnetic nanoparticles as a drug delivery system that enhance fungicidal activity of polyene antibiotics. Nanomedicine 12, 2395–2404. 10.1016/j.nano.2016.07.006 27464757

[B79] Odds F. C. Brown A. J. Gow N. A. R. (2003). Antifungal agents: mechanisms of action. Trends Microbiol. 11, 272–279. 10.1016/S0966-842X(03)00117-3 12823944

[B80] Pagels R. F. Prud'homme R. K. (2015). Polymeric nanoparticles and microparticles for the delivery of peptides, biologics, and soluble therapeutics. J. Control. Release 219, 519–535. 10.1016/j.jconrel.2015.09.001 26359125

[B81] Pandey R. Ahmad Z. Sharma S. Khuller G. K. (2005). Nano-encapsulation of azole antifungals: potential applications to improve oral drug delivery. Int. J. Pharm. 301, 268–276. 10.1016/j.ijpharm.2005.05.027 16023808

[B82] Pandit J. Garg M. Jain N. K. (2014). Miconazole nitrate bearing ultraflexible liposomes for the treatment of fungal infection. J. Liposome Res. 24, 163–169. 10.3109/08982104.2013.871025 24479833

[B83] Pardeike J. Weber S. Zarfl H. P. Pagitz M. Zimmer A. (2016). Itraconazole-loaded nanostructured lipid carriers (NLC) for pulmonary treatment of aspergillosis in falcons. Eur. J. Pharm. Biopharm. 108, 269–276. 10.1016/j.ejpb.2016.07.018 27449629

[B84] Patel N. R. Damann K. Leonardi C. Sabliov C. M. (2010). Itraconazole-loaded poly(lactic-co-glycolic) acid nanoparticles for improved antifungal activity. Nanomedicine (Lond). 5, 1037–1050. 10.2217/nnm.10.68 20874019

[B85] Patel N. R. Damann K. Leonardi C. Sabliov C. M. (2011). Size dependency of PLGA-nanoparticle uptake and antifungal activity against *Aspergillus flavus*. Nanomedicine (Lond). 6, 1381–1395. 10.2217/nnm.11.35 21651442

[B86] Peng H. S. Liu X. J. Lv G. X. Sun B. Kong Q. F. Zhai D. X.. (2008). Voriconazole into PLGA nanoparticles: improving agglomeration and antifungal efficacy. Int. J. Pharm. 352, 29–35. 10.1016/j.ijpharm.2007.10.009 18053659

[B87] Perez A. P. Altube M. J. Schilrreff P. Apezteguia G. Celes F. S. Zacchino S.. (2016). Topical amphotericin B in ultradeformable liposomes: formulation, skin penetration study, antifungal and antileishmanial activity *in vitro*. Colloids Surf. B Biointerfaces 139, 190–198. 10.1016/j.colsurfb.2015.12.003 26709977

[B88] Polvi E. J. Li X. O'Meara T. R. Leach M. D. Cowen L. E. (2015). Opportunistic yeast pathogens: reservoirs, virulence mechanisms, and therapeutic strategies. Cell. Mol. Life Sci. 72, 2261–2287. 10.1007/s00018-015-1860-z 25700837 PMC11113693

[B89] Prasad R. Shah A. H. Rawal M. K. (2016). Antifungals: Mechanism of Action and Drug Resistance, in Yeast Membrane Transporter Advances in Experimental Medicine and Biology, eds Ramos J. Sychrová H. Kschischo M. (Cham: Springer International Publishing), 327–349. 10.1007/978-3-319-25304-6_14 26721281

[B90] Qiu L. Hu B. Chen H. Li S. Hu Y. Zheng Y.. (2015). Antifungal efficacy of itraconazole-loaded TPGS-b-(PCL-ran-PGA) nanoparticles. Int. J. Nanomedicine 10, 1415–1423. 10.2147/IJN.S71616 25733833 PMC4337504

[B91] Reis J. (2015). Liposomal formulations of amphotericin B : differences according to the scientific evidence. Rev. Esp. Quimioter. 28, 275–281. 26621170

[B92] Rençber S. Karavana S. Y. Yılmaz F. F. Eraç B. Nenni M. Özbal S.. (2016). Development, characterization, and *in vivo* assessment of mucoadhesive nanoparticles containing fluconazole for the local treatment of oral candidiasis. Int. J. Nanomedicine 11, 2641–2653. 10.2147/IJN.S103762 27358561 PMC4912316

[B93] Ribeiro A. M. Souza A. C. O. Amaral A. C. Vasconcelos N. M. Jeronimo M. S. Carneiro F. P.. (2013). Nanobiotechnological approaches to delivery of DNA vaccine against fungal infection. J. Biomed. Nanotechnol. 9, 221–230. 10.1166/jbn.2013.1491 23627048

[B94] Saldanha C. A. Garcia M. P. Iocca D. C. Rebelo L. G. Souza A. C. O. Bocca A. L.. (2016). Antifungal activity of amphotericin B conjugated to nanosized magnetite in the treatment of paracoccidioidomycosis. PLoS Negl. Trop. Dis. 10:e0004754. 10.1371/journal.pntd.0004754 27303789 PMC4909273

[B95] Sanchez D. A. Schairer D. Tuckman-Vernon C. Chouake J. Kutner A. Makdisi J.. (2014). Amphotericin B releasing nanoparticle topical treatment of Candida spp. in the setting of a burn wound. Nanomedicine 10, 269–277. 10.1016/j.nano.2013.06.002 23770066

[B96] Sanglard D. Coste A. Ferrari S. (2009). Antifungal drug resistance mechanisms in fungal pathogens from the perspective of transcriptional gene regulation. FEMS Yeast Res. 9, 1029–1050. 10.1111/j.1567-1364.2009.00578.x 19799636

[B97] Serrano D. R. Lalatsa A. Dea-Ayuela M. A. Bilbao-Ramos P. E. Garrett N. L. Moger J.. (2015). Oral particle uptake and organ targeting drives the activity of amphotericin B nanoparticles. Mol. Pharm. 12, 420–431. 10.1021/mp500527x 25558881

[B98] Seyedmousavi S. Rafati H. Ilkit M. Tolooe A. Hedayati M. T. Verweij P. (2017). Systemic Antifungal Agents: Current Status and Projected Future Developments, in Methods in Molecular Biology, ed Lion T. (New York, NY: Springer New York), 107–139. 10.1007/978-1-4939-6515-1_5 27837500

[B99] Shapiro R. S. Robbins N. Cowen L. E. (2011). Regulatory circuitry governing fungal development, drug resistance, and disease. Microbiol. Mol. Biol. Rev. 75, 213–267. 10.1128/MMBR.00045-10 21646428 PMC3122626

[B100] Sheikh S. Ali S. M. Ahmad M. U. Ahmad A. Mushtaq M. Paithankar M.. (2010). Nanosomal amphotericin B is an efficacious alternative to ambisome for fungal therapy. Int. J. Pharm. 397, 103–108. 10.1016/j.ijpharm.2010.07.003 20621173

[B101] Shirkhani K. Teo I. Armstrong-James D. Shaunak S. (2015). Nebulised amphotericin B-polymethacrylic acid nanoparticle prophylaxis prevents invasive aspergillosis. Nanomedicine 11, 1217–1226. 10.1016/j.nano.2015.02.012 25791815 PMC4503863

[B102] Sil A. Andrianopoulos A. (2015). Thermally dimorphic human fungal pathogens–polyphyletic pathogens with a convergent pathogenicity trait. Cold Spring Harb. Perspect. Med. 5:a019794. 10.1101/cshperspect.a019794 25384771 PMC4526722

[B103] Sinha B. Mukherjee B. Pattnaik G. (2013). Poly-lactide-co-glycolide nanoparticles containing voriconazole for pulmonary delivery: *in vitro* and *in vivo* study. Nanomedicine 9, 94–104. 10.1016/j.nano.2012.04.005 22633899

[B104] Souza A. C. O. Nascimento A. L. de Vasconcelos N. M. Jerônimo M. S. Siqueira I. M. Santos R-L.. (2015). Activity and *in vivo* tracking of Amphotericin B loaded PLGA nanoparticles. Eur. J. Med. Chem. 95, 267–276. 10.1016/j.ejmech.2015.03.022 25827397

[B105] Stiufiuc R. Iacovita C. Stiufiuc G. Florea A. Achim M. Lucaciu C. M. (2015). A new class of pegylated plasmonic liposomes: synthesis and characterization. J. Colloid Interface Sci. 437, 17–23. 10.1016/j.jcis.2014.09.023 25310578

[B106] Takemoto K. (2006). Comparative study on the efficacy of AmBisome and Fungizone in a mouse model of pulmonary aspergillosis. J. Antimicrob. Chemother. 57, 724–731. 10.1093/jac/dkl005 16446374

[B107] Tang X. Dai J. Xie J. Zhu Y. Zhu M. Wang Z.. (2015a). Enhanced Antifungal Activity by Ab-Modified Amphotericin B-Loaded Nanoparticles Using a pH-Responsive Block Copolymer. Nanoscale Res. Lett. 10:969. 10.1186/s11671-015-0969-1 26061446 PMC4486495

[B108] Tang X. Jiao R. Xie C. Xu L. Huo Z. Dai J.. (2015b). Improved antifungal activity of amphotericin B-loaded TPGS-b-(PCL-ran-PGA) nanoparticles. Int. J. Clin. Exp. Med. 8, 5150–5162. 10.1016/j.ijbiomac.2014.10.014 26131089 PMC4483864

[B109] Tang X. Liang Y. Zhu Y. Xie C. Yao A. Chen L.. (2015c). Anti-transferrin receptor-modified amphotericin B-loaded PLA-PEG nanoparticles cure Candidal meningitis and reduce drug toxicity. Int. J. Nanomedicine 10, 6227–6241. 10.2147/IJN.S84656 26491294 PMC4599718

[B110] Tang X. Zhu H. Sun L. Hou W. Cai S. Zhang R.. (2014). Enhanced antifungal effects of amphotericin B-TPGS-b-(PCL-ran-PGA) nanoparticles *in vitro* and *in vivo*. Int. J. Nanomedicine 9:5403. 10.2147/IJN.S71623 25473279 PMC4247144

[B111] Tassel D. Madoff M. A. (1968). Treatment of Candida sepsis and Cryptococcus meningitis with 5-fluorocytosine. A new antifungal agent. JAMA 206, 830–832. 5695668

[B112] Tiyaboonchai W. Woiszwillo J. Middaugh C. R. (2001). Formulation and characterization of amphotericin B-polyethylenimine-dextran sulfate nanoparticles. J. Pharm. Sci. 90, 902–914. 10.1002/jps.1042 11458338

[B113] Trombino S. Mellace S. Cassano R. (2016). Solid lipid nanoparticles for antifungal drugs delivery for topical applications. Ther. Deliv. 7, 639–647. 10.4155/tde-2016-0040 27582235

[B114] Vanden Bossche H. Koymans L. Moereels H. (1995). P450 inhibitors of use in medical treatment: focus on mechanisms of action. Pharmacol. Ther. 67, 79–100. 7494862 10.1016/0163-7258(95)00011-5

[B115] Van de Ven H. Paulussen C. Feijens P. B. Matheeussen A. Rombaut P. Kayaert P.. (2012). PLGA nanoparticles and nanosuspensions with amphotericin B: potent *in vitro* and *in vivo* alternatives to Fungizone and AmBisome. J. Control. Release 161, 795–803. 10.1016/j.jconrel.2012.05.037 22641062

[B116] Van Slooten M. L. Boerman O. Romøren K. Kedar E. Crommelin D. J. Storm G. (2001). Liposomes as sustained release system for human interferon-gamma: biopharmaceutical aspects. Biochim. Biophys. Acta 1530, 134–145. 10.1016/S1388-1981(00)00174-8 11239816

[B117] Vieira D. B. Carmona-Ribeiro A. M. (2008). Cationic nanoparticles for delivery of amphotericin B: preparation, characterization and activity *in vitro*. J. Nanobiotechnology 6:6. 10.1186/1477-3155-6-6 18462496 PMC2408927

[B118] Vyas S. P. Gupta S. (2006). Optimizing efficacy of amphotericin B through nanomodification. Int. J. Nanomedicine 1, 417–432. 10.2147/nano.2006.1.4.417 17722276 PMC2676632

[B119] Walsh T. J. Finberg R. W. Arndt C. Hiemenz J. Schwartz C. Bodensteiner D.. (1999). Liposomal amphotericin B for empirical therapy in patients with persistent fever and neutropenia. National Institute of Allergy and Infectious Diseases Mycoses Study Group. N. Engl. J. Med. 340, 764–771. 10.1056/NEJM199903113401004 10072411

[B120] Wang L. Geng D. Su H. (2014). Safe and efficient pH sensitive tumor targeting modified liposomes with minimal cytotoxicity. Colloids Surf. B Biointerfaces 123, 395–402. 10.1016/j.colsurfb.2014.09.003 25438693

[B121] Wong-Beringer A. Jacobs R. A. Guglielmo B. J. (1998). Lipid formulations of amphotericin B: clinical efficacy and toxicities. Clin. Infect. Dis. 27, 603–618. 10.1086/514704 9770163

[B122] Xie J. L. Polvi E. J. Shekhar-Guturja T. Cowen L. E. (2014). Elucidating drug resistance in human fungal pathogens. Future Microbiol. 9, 523–542. 10.2217/fmb.14.18 24810351

[B123] Xu N. Gu J. Zhu Y. Wen H. Ren Q. Chen J. (2011). Efficacy of intravenous amphotericin B-polybutylcyanoacrylate nanoparticles against cryptococcal meningitis in mice. Int. J. Nanomedicine 6, 905–913. 10.2147/IJN.S17503 21720503 PMC3124396

[B124] Zhao M. Hu J. Zhang L. Zhang L. Sun Y. Ma N.. (2014). Study of amphotericin B magnetic liposomes for brain targeting. Int. J. Pharm. 475, 9–16. 10.1016/j.ijpharm.2014.08.035 25151436

[B125] Zhou W. Wang Y. Jian J. Song S. (2013). Self-aggregated nanoparticles based on amphiphilic poly(lactic acid)-grafted-chitosan copolymer for ocular delivery of amphotericin B. Int. J. Nanomedicine 8, 3715–3728. 10.2147/IJN.S51186 24106427 PMC3792006

[B126] Zia Q. Khan A. A. Swaleha Z. Owais M. (2015). Self-assembled amphotericin B-loaded polyglutamic acid nanoparticles: preparation, characterization and *in vitro* potential against Candida albicans. Int. J. Nanomedicine 10, 1769–1790. 10.2147/IJN.S63155 25784804 PMC4356689

[B127] Zonios D. I. Bennett J. E. (2008). Update on azole antifungals. Semin. Respir. Crit. Care Med. 29, 198–210. 10.1055/s-2008-1063858 18366001

